# Factors associated with ICU mortality and long-term outcomes in immunocompromised patients admitted to the intensive care unit for acute respiratory failure

**DOI:** 10.1186/s13613-025-01578-1

**Published:** 2025-10-30

**Authors:** Mélanie Métais, Jean-Pierre Frat, Stephan Ehrmann, Frédéric Pène, Maxens Decavèle, Nicolas Terzi, Gwenaël Prat, Maëlle Martin, Damien Contou, Arnaud Gacouin, Jeremy Bourenne, Christophe Girault, Christophe Vinsonneau, Jean Dellamonica, Guylaine Labro, Sébastien Jochmans, Alexandre Herbland, Jean-Pierre Quenot, Jérôme Devaquet, Dalila Benzekri, Stéphanie Ragot, Arnaud W. Thille, Rémi Coudroy, the FLORALI-IM study group and the REVA Research Network, Emmanuel Vivier, Saad Nseir, Gwenhaël Colin, Didier Thevenin, Giacomo Grasselli, David Bougon, Mona Assefi, Claude Guérin, Thierry Lherm, Achille Kouatchet

**Affiliations:** 1 Service de Médecine Intensive Réanimation, CHU de Poitiers, 2 rue de la Milétrie, Poitiers, F-86000 France; 2 IS-ALIVE Research Group, INSERM CIC 1402, Université De Poitiers, Poitiers, France; 3 Centre d’étude des Pathologies Respiratoires, CHRU de Tours, CRICS-TriggerSEP F- CRIN Research Network, INSERM U1100, Université de Tours, Médecine Intensive Réanimation, CIC 1415, Tours, France; 4 Médecine Intensive & Réanimation, APHP Centre, Hôpital Cochin, Université de Paris, Paris, France; 5 Service Médecine Intensive et Réanimation (Département R3S), Groupe Hospitalier Universitaire APHP-Sorbonne Université, site Pitié-Salpêtrière, Paris, France; 6 Sorbonne Université, INSERM, UMRS1158 Neurophysiologie Respiratoire Expérimentale et Clinique, Paris, France; 7 University of Grenoble Alpes, INSERM U1300, Grenoble, France; 8 Medical Intensive Care Unit, Medical Intensive Care Unit, Grenoble France, University of Rennes, Rennes, France; 9 Médecine Intensive Réanimation, CHU de Brest, Brest, France; 10 Médecine Intensive Réanimation, CHU de Nantes, Nantes, France; 11 Service de Réanimation Polyvalente, Centre Hospitalier Victor Dupouy, Argenteuil, France; 12 Service des Maladies Infectieuses et Réanimation Médicale, CHU de Rennes, Hôpital Ponchaillou, Rennes, France; 13 Médecine Intensive Réanimation, Réanimation des Urgences, CHU La Timone 2, Aix-Marseille Université, Marseille, France; 14 Department of Medical Intensive Care, Univ Rouen Normandie, CHU Rouen, Rouen, GRHVN UR 3830, F-76000 France; 15 Service de Réanimation, Centre hospitalier de Béthune, Beuvry, France; 16 Médecine Intensive Réanimation, Centre Hospitalier Universitaire de Nice, Nice, France; 17 UR2CA Unité de Recherche Clinique Côte d’Azur, Université Cote d’Azur, Nice, France; 18 Medical Intensive Care Unit, University Hospital, Besançon, France; 19 Service de Réanimation, Centre hospitalier Sud-Ile-de France, Melun, France; 20 Service De Réanimation, Centre Hospitalier Saint Louis, La Rochelle, France; 21 Service de Médecine Intensive-Réanimation, CHU Dijon-Bourgogne Équipe Lipness, Centre De Recherche INSERM UMR1231 et LabEx LipSTIC, Université de Bourgogne-Franche Comté, Dijon, France; 22 INSERM, CIC 1432, Module Épidémiologie Clinique, université de Bourgogne-Franche Comté, Dijon, France; 23 Service de Réanimation Polyvalente, Hôpital Foch, Suresnes, France; 24 Médecine Intensive Réanimation, Centre Hospitalier Régional D’Orléans, Orléans, France; 25 INSERM CIC 1402, Université de Poitiers, Biostatistics, France

**Keywords:** Immunosuppression, Acute respiratory failure, Intensive care unit, Mortality

## Abstract

**Background:**

Mortality of immunocompromised patients is particularly high in intensive care units (ICUs) and mainly depends on severity at admission. Moreover, mortality is also high during the months following ICU discharge. The reasons for these poor outcomes after ICU discharge have not been adequately studied.

**Research question:**

We hypothesized that the factors associated with poor outcomes after ICU discharge of immunocompromised patients would be different from those associated with in-ICU mortality.

**Study design and methods:**

This is a post-hoc analysis of a multicenter clinical trial comparing two noninvasive oxygenation strategies in immunocompromised patients admitted to ICU for acute hypoxemic respiratory failure. Multivariable analyses were performed to determine early factors (i.e within 6 h of admission) associated with in-ICU mortality, as well as factors associated with poor functional outcomes (i.e death or survival with poor performance status) at 6 months, only in ICU survivors.

**Results:**

Among the 299 patients analyzed, the mortality rate was 31% (94 patients) in the ICU and 49% at 6 months (146 patients). Solid cancer (adjusted odds ratio 2.92 [95% confidence interval, 1.22–7.28]), severity SOFA score at admission (aOR 1.29 [1.14–1.48]), the extent of pulmonary infiltrates on chest X-ray (aOR 1.57 [1.17–2.15]) and increased discomfort one hour after initiation of noninvasive respiratory support (aOR 2.08 [1.12–3.85]) were independently associated with in-ICU mortality. Out of the 202 ICU survivors whose performance status was reported, solid cancer (aOR 3.03 [1.33–9.09]) and poor performance status before ICU admission (aOR 2.43 [1.03–5.88]) were both associated with poor outcome at 6 months, independently from the decision to forgo life-sustaining therapies (aOR 5.88 [2.17–20.00]).

**Interpretation:**

Whereas in-ICU mortality of immunocompromised patients with acute respiratory failure was mainly driven by severity, poor outcomes at 6 months were mainly driven by performance status before ICU admission. Solid cancer was independently associated with both poor short as well as longer-term outcomes.

*Trial registration* Clinical trial registration: NCT04227639

**Supplementary Information:**

The online version contains supplementary material available at 10.1186/s13613-025-01578-1.

## Introduction

Immunosuppression is defined as a dysfunction of the immune system, whether primary or secondary, resulting from a medical condition such as solid cancer, hematologic malignancy, human immunodeficiency virus, or immunosuppressive drugs [1]. The prevalence of immunosuppression has been steadily increasing in the recent years, due primarily to the rising incidence of cancer and the improved survival of these patients, stemming from earlier detection of diseases and therapeutic advances [2]. Therefore, it can be anticipated that a considerable proportion of patients will live several years with various types and degrees of immunosuppression, exposing them to an increased risk of developing severe life-threatening infections [3]. As a result, immunocompromised patients account for 15% to 25% of all patients admitted to intensive care units (ICUs) [4].

Acute respiratory failure is the main reason for ICU admission in this subset of patients [5]. Their mortality in the ICU is high, approximating 30% in overall [6], and may exceed 50% in those requiring invasive mechanical ventilation [7]. In addition, their mortality after ICU discharge continues to increase, reaching about 50% at six months and over 60% at one year after ICU admission [8, 9]. However, risk factors for mortality among ICU survivors have not been adequately studied. Indeed, most of the studies assessing factors associated with long-term mortality in immunocompromised patients admitted to the ICU have to a large extend based their analyses on factors associated with ICU mortality, such as the severity of organ failure [8, 9]. Additionally, although they are of major importance when selecting an adequate therapeutic option in cancer or hematology patients [8, 10, 11], the factors influencing functional status after discharge have only rarely been assessed in immunocompromised patients admitted to the ICU for acute hypoxemic respiratory failure [12]. Accounting for the functional status assessment of immunocompromised patients after ICU discharge would be of primary importance in order to better understand which patients would benefit from ICU admission or readmission.

We hypothesized that among immunocompromised patients admitted to an ICU for acute respiratory failure, the factors associated with ICU mortality outcomes differed from those associated with poor outcomes after ICU discharge.

## Methods

### Study design

We performed an unplanned post-hoc analysis of a randomized controlled trial conducted in 29 centers in France and in Italy comparing two noninvasive oxygenation strategies in immunocompromised patients admitted to the ICU for acute hypoxemic respiratory failure [7]. The protocol was approved by the Ethics Committee Ouest III (Poitiers, France) for French centers and by the local ethics committee for the Italian center. Informed consent from patients or their surrogate was obtained orally, with a written record maintained by the investigator.

### Patients

Adult (≥ 18 years old) immunocompromised patients admitted to the ICU for acute hypoxemic respiratory failure defined as a respiratory rate ≥ 25 breaths per minute and PaO_2_/FiO_2_ ratio ≤ 300 mmHg while spontaneously breathing with standard oxygen (oxygen flow rate ≥ 10 L/min), with high-flow nasal oxygen therapy or with noninvasive ventilation were included. Immunosuppression was defined by one of the following criteria: hematological malignancy (active or remitting < 5 years), allogenic stem cell transplantation within the previous 5 years, active or relapsing solid cancer, leucopenia < 1 G/L or neutropenia ≤ 0,5 /induced by chemotherapy, solid organ transplantation, acquired immunodeficiency syndrome, systemic steroids ≥ 0.5 mg/kg per day of prednisone equivalent for at least 3 weeks, or immunosuppressive or immunomodulatory drugs. The main exclusion criteria were patients who could strongly benefit from noninvasive ventilation (hypercapnia defined as PaCO_2_ > 50 mmHg-, acute-on-chronic respiratory failure, cardiogenic pulmonary edema, surgery less than 7 days before), shock, impaired consciousness, urgent need for intubation, and Do Not Intubate order at time of inclusion. Additionally, for the current analysis, we excluded patients in whom Eastern Cooperative Oncology Group (ECOG) performance status at 6 months after randomization was missing.

### Classification of immunosuppression subgroups

Patients were classified according to the underlying cause of immunosuppression in 3 subgroups: (1) active or remitting for less than 5 years hematological malignancy whatever the treatment (chemotherapy alone, autologous stem cell transplantation, allogeneic stem cell transplantation), (2) active solid cancer and (3) other causes (i.e. acquired immune deficiency syndrome, solid organ transplant recipient or immunosuppressive drugs for connective tissue disease). For patients with more than one cause of immunosuppression (for instance a solid organ transplant recipient who developed lymphoma), the most recent disease that led to ICU admission for acute hypoxemic respiratory failure was considered as the main cause of immunosuppression.

### Data collection

Demographic characteristics (age, sex, comorbidities, cause of immunosuppression, ECOG performance status before ICU admission), cause of acute respiratory failure, severity scores including Simplified Acute Physiology Score (SAPS) II at ICU admission [13] and Sequential Organ Failure Assessment (SOFA) at admission [14] clinical characteristics and discomfort score (by means of a 100 mm visual analogue scale ranging from 0 - “no discomfort” – to 100 – “maximal imaginable discomfort”) before treatment initiation and after one hour of treatment, presence of bilateral infiltrates and the number of quadrants with infiltrates on chest X- ray at inclusion according to the investigator, treatments during ICU stay (need for intubation, dialysis, or chemotherapy, and decision to forgo life-sustaining therapies) and outcomes (mortality at day 28, at 6 months, and ECOG performance status at 6 months) were prospectively collected.

### Study outcomes

Poor outcome at 6 months was defined as death or survival with ECOG performance status of 3 or 4 at 6 months after admission among ICU survivors.

### Statistical analysis

Continuous variables were expressed as mean ± standard deviation (SD) or median (25–75th percentiles) depending on their distribution and compared using the Student t-test or the Mann-Whitney *U* test, as appropriate. Categorical variables were expressed as number (percentage) and compared using the Chi-squared test or Fisher test, as appropriate. A first stepwise backwards logistic regression model was computed to identify early variables (i.e. occurring within the first 6 h after admission) associated with ICU mortality in the overall population. Intubation within the first 24 h after ICU admission and SAPS II were not included in the model despite their strong association with ICU mortality [6, 13] because these variables require at least 24 h after ICU admission to be considered, hampering their clinical utility as early markers. A second stepwise backward logistic regression model was computed to identify the variables associated with poor outcomes at 6 months in the subset of ICU survivors. Variables included in the multivariable analysis included noncollinear variables associated with poor outcomes with a p value < 0.20 in univariate analysis. Variables known to be associated with poor outcomes and the noninvasive oxygenation strategy allocated were also forced in the models. Statistical analyses were conducted by using R software version 4.3.2 (R Foundation for Statistical Computing; https://www.R-project.org). Two-tailed p values < 0.05 were considered significant.

## Results

Among the 299 immunocompromised patients included in the seminal study, malignancy (either hematological or solid) was the cause of immunosuppression in 74% of cases (222 patients). Overall, ECOG performance status before ICU admission was 1 ± 1 in mean, including 16% of patients (49 out of 299) with a poor score of 3 or 4. At admission, severity SAPS-II and SOFA scores were 45 ± 16 and 6 ± 3, respectively. At inclusion, the respiratory rate was 31 ± 5 breaths/min, PaO_2_/FiO_2_ ratio 147 ± 56 mmHg, and 74% of patients (223 out of 299) had bilateral infiltrates on chest X-ray. Characteristics of patients according to the immunosuppression subgroup is displayed in Additional Table 1.

**Table 1:** Univariate analysis of factors associated with ICU mortality

Variables	Death in the ICU (*n* = 94)	Alive at ICU discharge (*n* = 205)	*p *value
Baseline characteristics at admission			
Age, years	65 ± 12	63 ± 12	0.111
Male sex, n (%)	62 (66%)	130 (63%)	0.767
Body mass index, kg/m^2^	25 ± 5.2	25 ± 5.8	0.670
Body mass index < 18.5 kg/m^2^, n (%)	6 (6.4%)	19 (9.3%)	0.566
Simplified Acute Physiology score II	51 ± 20	43 ± 14	**< 0.001**
ECOG performance status 3 or 4, n (%)	20 (21%)	29 (14%)	0.168
Charlson comorbidity score	3.6 ± 2.6	3.4 ± 2.3	0.585
Underlying condition, n (%)			
Type of immunosuppression			0.085
Hematological malignancy	47 (50%)	103 (50%)	
Solid cancer	29 (31%)	43 (21%)	
Other cause ^A^	18 (19%)	59 (29%)	
Characteristics at inclusion			
SOFA score	6.8 ± 3.0	5.7 ± 2.5	**0.002**
SOFA score without respiratory item	3.6 ± 3.0	2.7 ± 2.4	**0.012**
Need for norepinephrine, n (%)	10 (11%)	8 (3.9%)	**0.044**
Thrombocytopenia, n (%)	12 (13%)	32 (16%)	0.639
Respiratory rate, breaths/min	32 ± 5	31 ± 5	0.072
pH, units	7.43 ± 0.09	7.44 ± 0.06	0.406
PaO_2_/FiO_2_, mmHg	134 ± 51	154 ± 58	**0.003**
PaCO_2_, mmHg	34 ± 6	34 ± 6	0.388
Discomfort score, mm ^B^	46 ± 27	45 ± 28	0.892
Bilateral infiltrates on chest X-ray, n (%)	75 (80%)	148 (72%)	0.209
Number of quadrants with infiltrates on chest X-ray	3.1 ± 1	2.8 ± 1.1	**0.014**
1 h after treatment initiation			
Randomization in the noninvasive ventilation arm, n (%)	49 (52%)	96 (47%)	0.468
Respiratory rate, breaths/min	30 ± 8	27 ± 7	**0.023**
pH, units	7.42 ± 0.10	7.45 ± 0.06	0.067
PaO_2_/FiO_2_, mmHg	146 ± 73	183 ± 92	**0.001**
PaCO_2_, mmHg	35 ± 6	34 ± 6	0.640
Discomfort score, mm^C^	45 ± 29	39 ± 27	0.129
Change between inclusion and H1, n (%)			
Increased respiratory rate	37 (39%)	70 (34%)	0.485
Increased PaO_2_/FiO_2_	41 (44%)	108 (53%)	0.181
Increased PaCO_2_	51 (54%)	98 (48%)	0.181
Increased discomfort score^C^	46 (49%)	69 (34%)	**0.045**

Qualitative variables are expressed in number (percentage), quantitative variables are expressed in mean ± standard deviation or median [25th−75thpercentile] according to their distribution.

ECOG: Eastern Cooperative Oncology Group; SOFA: Sequential organ failure assessment; PaO_2_: partial pressure of arterial oxygen; FiO_2_: fraction of inspired oxygen, PaCO_2_: partial pressure of arterial carbon dioxide; H1: 1 h after randomization.

^A^ Acquired immune deficiency syndrome, solid organ transplant or immunosuppressive drugs for connective tissue disease.

Discomfort score was assessed using a 100 mm visual analogue scale ranging from no discomfort (0) to maximum imaginable discomfort (100).

^C^ H1 refers to one h after randomization.

^D^ assessed in 250 patients.

**Fig. 1:**
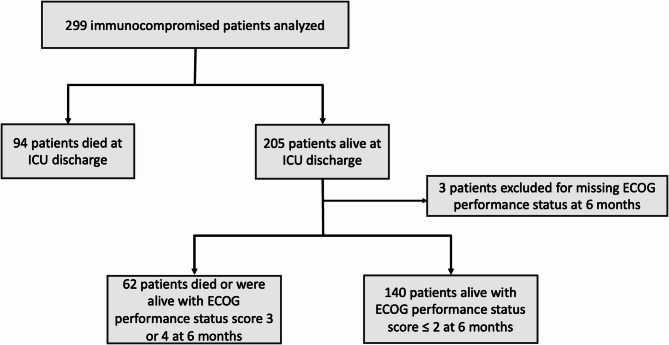
Flow chart of patients included in the analysis

Mortality in ICU and at 6 months was 31% (94 patients) and 49% (146 patients), respectively. Among the 205 ICU survivors, and after excluding three patients whose ECOG performance status was missing at 6 months (Additional Table 2.), 31% had a poor outcome at 6 months (62 out of 202 patients), including 52 patients who died after ICU discharge (26%) and 10 patients who were alive with ECOG performance status 3 or 4 (5%) at 6 months (Fig. 1).

**Table 2:** Univariate analysis of factors associated with poor outcome at 6 months among ICU survivors

Variables	Died or alive with ECOG 3 or 4 at 6 months (*n* = 62)	Alive with ECOG ≤ 2 at 6 months (*n* = 140)	*p *value
Baseline patient characteristics			
Age, years	62 ± 12	63 ± 13	0.614
Sex, male, n (%)	37 (60%)	90 (64%)	0.640
Body mass index, kg/m^2^	24 ± 5.8	25 ± 5.8	0.211
Body mass index < 18.5 kg/m^2^, n (%)	10 (16%)	9 (6%)	0.058
Simplified Acute Physiology score II	43 ± 14	42 ± 14	0.621
ECOG performance status 3 or 4, n (%)	14 (23%)	15 (11%)	**0.045**
Charlson comorbidity score	3.8 ± 2.7	3.2 ± 2.0	0.088
Underlying condition, n (%)			
Type of immunosuppression			**< 0.001**
Hematological malignancy	28 (45%)	74 (53%)	
Solid cancer	23 (37%)	19 (14%)	
Other ^a^	11 (18%)	47 (34%)	
During intensive care unit stay			
Randomization in the noninvasive ventilation arm, n (%)	28 (45%)	67 (48%)	0.840
ARDS according to the new definition at randomization, n (%)			0.999
No or mild ARDS	26 (42%)	58 (41%)	
Moderate or severe ARDS	36 (58%)	82 (59%)	
Bronchoalveolar lavage, n (%)	20 (32%)	57 (41%)	0.670
CT-scan, n (%)	37 (60%)	91 (65%)	0.572
Change in immunosuppressive drug regimen, n (%)	18 (29%)	36 (26%)	0.750
Renal replacement therapy, n (%)	7 (11%)	12 (8.6%)	0.727
Need for norepinephrine within 3 days after randomization, n (%)	8 (13%)	11 (7.9%)	0.365
Intubation, n (%)	16 (26%)	43 (31%)	0.589
Duration of mechanical ventilation, days	10 ± 7	12 ± 9	0.433
Reintubation, n (%)	1 (6%)	3 (7%)	1.000
Decision to forgo life-sustaining therapies, n (%)	16 (26%)	6 (4.3%)	**< 0.001**
Cause of respiratory failure, n (%)			
Microbiologically documented lung infection	24 (39%)	79 (56%)	**0.030**
Specific	11 (18%)	13 (9.3%)	0.140
Toxic cause	3 (4.8%)	8 (5.7%)	0.999
Cardiogenic pulmonary edema	2 (3.23%)	9 (6.4%)	0.556
Miscellaneous	5 (8.1%)	12 (8.6%)	0.999
No diagnosis	15 (24%)	18 (13%)	0.071
Short-term outcomes			
Length of stay in the ICU, days	7 [5–12]	8 [5–14]	0.122
Length of stay in the hospital, days	22 [14–36]	19 [13–31]	0.596
Length of hospital stay after ICU discharge, days	12 [5–22]	10 [6–18]	0.542

Qualitative variables are expressed in number (percentage), quantitative variables are expressed in mean ± standard deviation or median [25th−75th percentile] according to their distribution

ECOG: Eastern Cooperative Oncology Group; ARDS: acute respiratory distress syndrome; CT-scan: computed tomography scan; ICU: intensive care unit

^a^ Acquired immune deficiency syndrome, solid organ transplant or immunosuppressive drugs for connective tissue disease

### Factors associated with ICU mortality

**Fig. 2:**
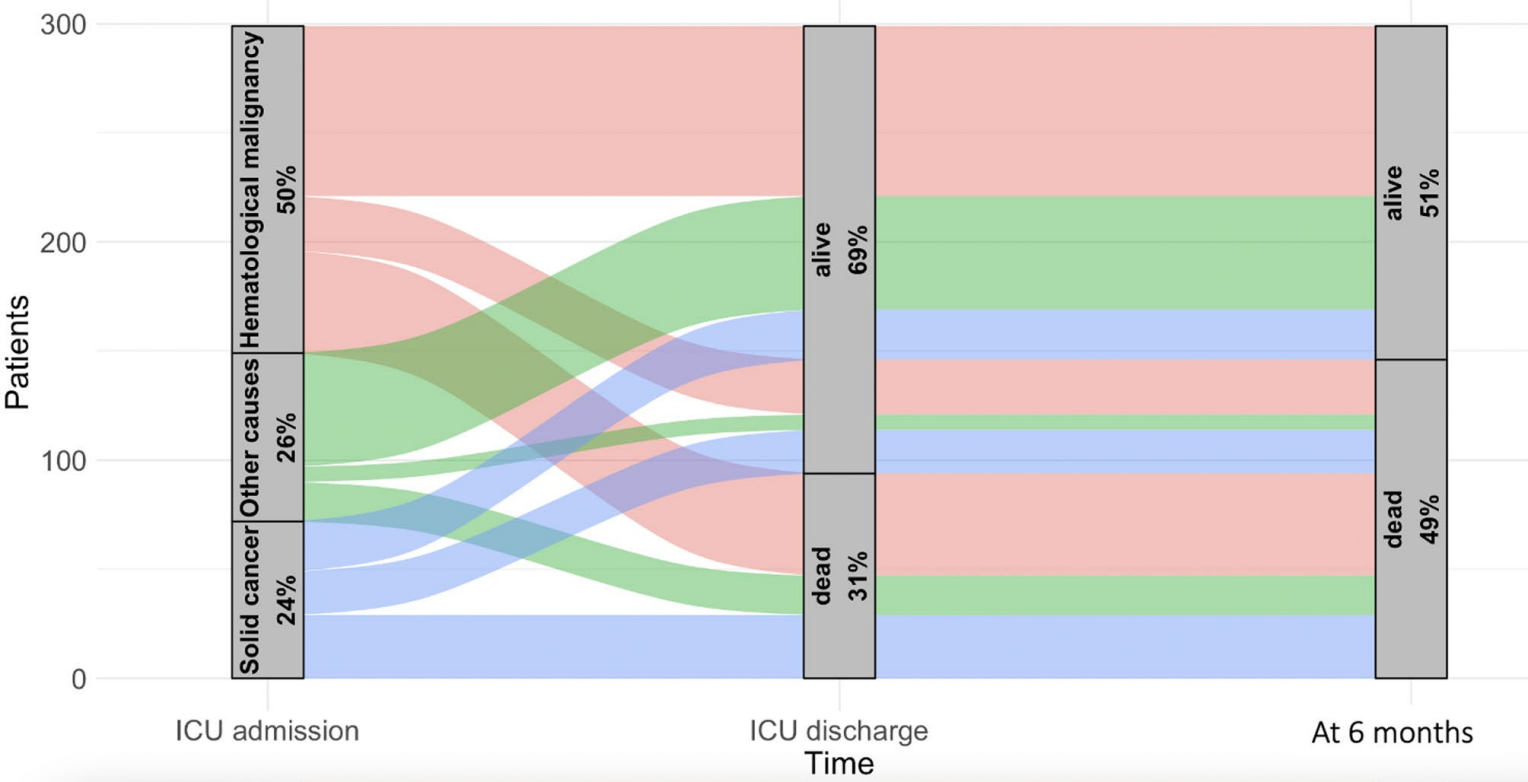
Alluvial plot of the trajectories of immunocompromised patients admitted to the ICU for acute respiratory failure according to their subtype of immunosuppression, up to ICU discharge and 6 months

Using univariate analysis, patients who died in the ICU were more likely to have higher severity scores, a higher number of quadrants with infiltrates on chest X-ray, higher respiratory rate and more severe hypoxemia at admission. Additionally, they were more likely to have an increased in discomfort score after treatment initiation than patients who survived in the ICU (Table 1 and Fig. 2).

**Fig. 3:**
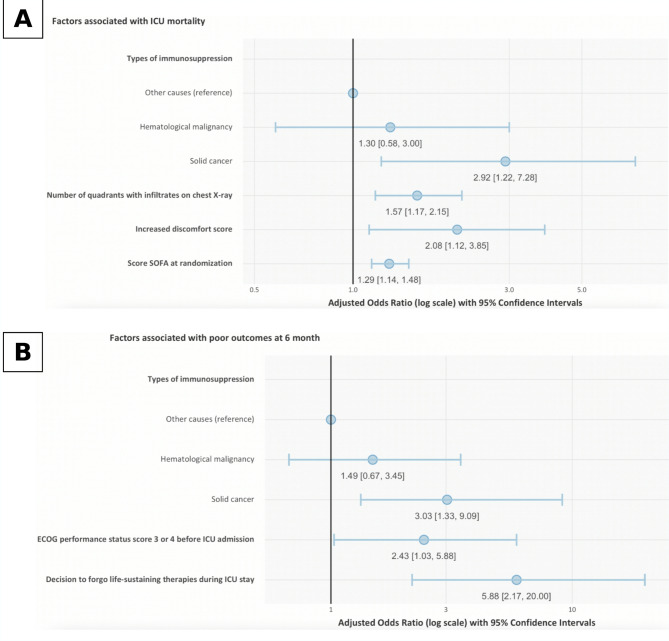
Forrest plot of factors independently associated with ICU mortality (panel** A**) and poor outcome at 6 months among ICU survivors (Panel** B**). Panel **A**: Variables included in the backward stepwise logistic regression model were age, ECOG performance status 3 or 4 before ICU admission, solid cancer, SOFA score at inclusion, number of quadrants with infiltrates on chest X-ray, respiratory rate at inclusion, respiratory rate and pH 1 h after treatment initiation, and increased PaO_2_/FiO_2_, PaCO_2_ and discomfort score between inclusion and 1 h after treatment initiation. The noninvasive oxygenation strategy allocated in the seminal study was forced in the model and interaction between variables was tested. (Hosmer-Lemeshow goodness-of-fit test: *p* = 0.41) Panel** B**: Variables included in the backward stepwise logistic regression model were ECOG performance status 3 or 4 before ICU admission, body mass index < 18.5 kg/m^2^, solid cancer and patients with other causes of immunosuppression, microbiologically documented lung infection, no etiological diagnosis of respiratory failure, decision to forgo life-sustaining therapies during ICU stay, and ICU length of stay in the ICU. Intubation was forced in the model. The noninvasive oxygenation strategy allocated in the seminal study was forced in the model and interaction between variables was tested. (Hosmer-Lemeshow goodness-of-fit test: *p* = 0.86)

Using multivariable analysis, solid cancer, radiographic severity defined by the number of quadrants with pulmonary infiltrates on chest X-ray and increased discomfort score after treatment initiation were associated with ICU mortality, independently from severity indicated by SOFA score (Fig. 3A).

### Factors associated with poor outcome at 6 months among ICU survivors

Using univariate analysis, patients with poor outcome after ICU discharge (i.e. who died or who were alive with an ECOG performance status of 3 or 4 at 6 months) were more likely to have solid cancer or a cause of immunosuppression other than hematologic disease. More patients with a poor outcome at 6 months had already ECOG performance status of 3 or 4 before ICU admission, decision to forgo life-sustaining therapies during ICU stay and to have no etiological diagnosis of respiratory failure than patients with good outcome at 6 months (Table 2 and Fig. 2).

Using multivariable analysis, solid cancer and ECOG performance status 3 or 4 before ICU admission were associated with poor outcome at 6 months, independently from the decision to forgo life-sustaining therapies (Fig. 3B).

## Discussion

In this post-hoc analysis of a randomized trial including immunocompromised patients admitted to an ICU for acute hypoxemic respiratory failure, severity SOFA score at admission, solid cancer, radiographic severity and increased discomfort after treatment initiation were independently associated with ICU mortality. Among admitted patients, ICU mortality was 31%. Of those discharged from the ICU, 31% either died before 6 months or had poor performance status at that time point.

After ICU discharge, solid cancer, ECOG performance status 3 or 4 before ICU admission and decision to forgo life-sustaining therapies during ICU stay were the three variables independently associated with poor outcomes at 6 months.

In our study, in-ICU mortality was 31%, in keeping with the 32% in-ICU mortality rate reported by in a large-scale prospective observational international cohort study including 1611 immunocompromised patients similar to ours (52% of patients with hematological malignancy and 35% with solid cancer) [6]. Overall mortality rate at 6 months was 49% which is also close to the 47% mortality rate reported in a retrospective study including 366 patients with hematological malignancy [15], and to the 55% mortality rate reported in a multicenter observational study including 449 patients with lung cancer [8]. All in all, these findings reinforce the external validity of our findings.

In addition to the severity score assessed at admission, we found that solid cancer, radiographic severity and increased discomfort after treatment initiation were three independent predictors of death in the ICU. Severity of organ failure has been extensively reported as a risk factor for ICU mortality in patients with hematological malignancy [16], lung cancer [8, 17], and in a mixed population of immunocompromised patients admitted to an ICU for acute respiratory failure [6]. Although scarcely compared in the literature, mortality of patients with solid cancer has been shown to be higher than that of patients with hematological malignancy [18, 19]. Unfortunately, due to the lack of power of our study, we were not able to compare outcomes according to types of solid cancer, a major determinant of short-term prognosis [20]. Likewise, the number of quadrants with pulmonary infiltrates on chest X-ray, indicating the radiologic severity of the respiratory disease, has previously been associated with increased risk of intubation or death in immunocompromised patients with acute respiratory failure [21, 22]. Interestingly, increased discomfort score one hour after initiation of noninvasive oxygenation strategy, a simple tool to use at the bedside, was independently associated with ICU mortality. This discomfort score may, at least partially, reflect dyspnea, an independent risk factor for noninvasive respiratory support failure in *de novo* respiratory failure [23]. Dyspnea is also associated with increased respiratory drive in mechanically ventilated patients, which is itself associated with poor outcomes [24]. Additionally, heterogeneity of treatment response according to dyspnea has been described in COVID-19 patients treated with noninvasive respiratory support [25]. However, whether personalization of noninvasive respiratory support changes patients’ dyspnea, discomfort and outcomes, or whether personalization of noninvasive respiratory support based on patient’s dyspnea or comfort changes outcomes remains to be tested. Unlike a previous study, poor functional status before ICU admission was not associated with ICU mortality [26]. This finding may be explained by differences in the populations analyzed (mostly non-small cell lung cancer vs. a mixed case of immunocompromised patients), by differences in the prevalence of patients with poor performance status before ICU admission (32% vs. 16% in our study) [26], and by the lack of data regarding the impact of functional status on outcomes outside cancer. All in all, in immunocompromised patients admitted to an ICU for acute hypoxemic respiratory failure, factors associated with in-ICU mortality were related to the underlying cause of immunosuppression, to the severity of the acute disease, and to the response to noninvasive respiratory support. Performance status before ICU admission may not be a good predictor of ICU mortality in this setting.

Regarding ICU survivors, we found that solid cancer and poor performance status before ICU admission were associated with poor outcomes at 6 months, independently from the decision to forgo life-sustaining therapies during ICU stay. The decision to forgo life-sustaining therapies is more frequent in immunocompromised than in immunocompetent patients [27]. A few studies have reported that the decision to forgo life-sustaining therapies was associated with poor long-term outcomes in ICU survivors with solid cancer [26, 28]. We confirm that this decision is also an independent predictor of poor long-term outcomes in a mixed-case population of immunocompromised patients. As for ICU mortality, solid cancer was still associated with high mortality after ICU discharge, up to 73% at 12 months according to a recent retrospective single center study [29]. Here, we confirm that solid cancer is a strong predictor of poor outcome at 6 months among ICU survivors. Likewise, poor functional status before ICU admission is a well-established risk factor for long-term mortality in ICU survivors with solid cancer [26, 28], likely because patients with poor performance status are less able to resume cancer treatments [29]. This finding reinforces the importance of interventions before ICU admission to improve cancer patients’ general condition. Interestingly, severity scores at ICU admission and organ failure during ICU stay, such as the need for intubation, were not independently associated with poor outcomes at 6 months among ICU survivors. All in all, our findings suggest that the decision for ICU readmission for immunocompromised patients should be more driven by the functional status before the index ICU admission, than by the intensity of organ failure during the index ICU stay.

Some limitations must be acknowledged. First, the randomized design of the seminal study, with patients selected based on strict inclusion and exclusion criteria, and the unplanned nature of the analysis could have led to selection bias and may have limited the generalizability of our results. However, the baseline characteristics and outcomes of patients are similar to that reported in the largest international observational study [6]. Second, variables included in the logistic regression models were based on data collected in the seminal study. We cannot exclude that data collected at other early timepoints, ECOG performance status at ICU and hospital discharge [29], timing of the decision to forgo life-sustaining therapies, treatment resumption after ICU discharge for cancer patients [26, 30] would have led to different findings. Third, the selection of variables included in the logistic regression models is debatable. For instance, the need for intubation was not included in the analysis of variables associated with ICU mortality because it occurs later than 24 h after ICU admission in about half of patients admitted to the ICU for acute respiratory failure and treated with first-line noninvasive oxygenation strategies [7, 31]. Likewise, SAPS II was not included in the model because its calculation requires 24 h of hindsight after ICU admission [13]. We rather chose early easy-to-assess variables that may be helpful for the clinician when deciding whether or not a patient could benefit from ICU admission. Last, data were collected at inclusion and not at ICU admission. However, the time elapsed between ICU admission and inclusion was very short (less than 15 min in median), suggesting that data collected at inclusion correspond to data at ICU admission.

## Conclusion

In immunocompromised patients admitted to the ICU for acute respiratory hypoxemic failure, solid cancer and increased patient discomfort after initiation of noninvasive respiratory support were associated with ICU mortality, independently from the clinical and radiographic severity. After ICU discharge, solid cancer was still associated with poor outcome at 6 months (i.e. death or survival with poor ECOG performance status of 3 or 4) independently of ECOG performance status before ICU admission and the decision to forgo life-sustaining therapies during ICU stay.

## Supplementary Information

Supplementary Material 1

## Data Availability

The data sets used and/or analyzed during the current study are available from the corresponding author upon reasonable request.

## Abbreviations

ECOG: Eastern cooperative oncology group
ICU: Intensive care unit
FiO_2_: Fraction of inspired dioxygen
PaO_2_: Partial pressure of arterial dioxygen
PaCO_2_: Partial pressure of arterial carbon dioxide
SAPSII: Simplified acute physiology score II
SOFA: Sequential organ failure assessment
SpO_2_: Pulse oximetry
ARDS: Acute respiratory distress syndrome

## Declarations

### **Prior abstract presentations**

Some of the results of this study have been presented as oral communications at the French Intensive Care Society meeting (Congrès Réanimation) in June 2024
