# Supplementary material for: Factors associated with ICU mortality and long-term outcomes in immunocompromised patients admitted to the intensive care unit for acute respiratory failure
Source: Ann Intensive Care. 2025 Oct 30;15:175. doi: 10.1186/s13613-025-01578-1 (PMC12575889; doi:10.1186/s13613-025-01578-1)
Supplement: Supplementary file 1 — Supplementary Material 1 [file 13613_2025_1578_MOESM1_ESM.docx]

**Additional material**

**Additional Table 1. Characteristics of patients according to the type of immunosuppression.**

| **Variables** | **Hematological malignancy**  **(n=150)** | **Solid cancer**  **(n=72)** | **Other cause**  **(n=77)** | **P value** |
| --- | --- | --- | --- | --- |
| **Baseline characteristics at admission** |  |  |  |  |
| Age, y | 65 ± 11 | 62 ± 12 | 60 ± 15 | **0.015** |
| Male sex, n (%) | 95 (63%) | 52 (72%) | 45 (58%) | 0.204 |
| Body mass index, kg/m^2^ | 26 ± 5 | 24 ± 5 | 25 ± 7 | **0.011** |
| Body mass index < 18,5 kg/m^2^, n (%) | 4 (3%) | 12 (17%) | 9 (12%) | **0.001** |
| Simplified Acute Physiology score II | 48 ± 15 | 43 ± 15 | 42 ± 18 | **0.005** |
| ECOG performance status 3 or 4, n (%) | 24 (16%) | 12 (17%) | 13 (17%) | 0.983 |
| Charlson comorbidity score | 2.6 ± 1.5 | 5.3 ± 2.6 | 3.4 ± 2.7 | **<0.001** |
| **Characteristics at Inclusion** |  |  |  |  |
| SOFA score | 6.8 ± 2.9 | 5.0 ± 2.1 | 5.5 ± 2.5 | **<0.001** |
| SOFA score without respiratory item | 3.8 ± 2.8 | 1.8 ± 2.0 | 2.4 ±2.3 | **<0.001** |
| Need for norepinephrine, n (%) | 11 (7%) | 3 (4%) | 4 (5%) | 0.610 |
| Thrombocytopenia, n (%) | 25 (17%) | 12 (17%) | 7 (9%) | 0.271 |
| Respiratory rate, breaths/min | 31 ± 5.3 | 31 ± 5.3 | 33 ± 6.1 | 0.145 |
| pH, units | 7.45 ± 0.07 | 7.44 ±0.07 | 7.43 ±0.07 | 0.305 |
| PaO_2_/FiO_2_, mmHg | 154 ± 59 | 131 ± 48 | 150 ± 57 | **0.013** |
| PaCO_2_, mmHg | 34 ± 6.1 | 36 ± 6.2 | 33 ± 5.5 | **0.003** |
| Discomfort score, mm | 48 ± 29 | 42 ± 27 | 45 ± 28 | 0.381 |
| Bilateral infiltrates on chest X-ray, n (%) | 107 (71%) | 56 (77%) | 60 (78%) | 0.433 |
| Number of quadrants with infiltrates on chest X-ray | 2.9 ± 1.1 | 2.8 ± 1.1 | 3.1 ± 1.1 | 0.288 |
| **1h after treatment initiation** |  |  |  |  |
| Randomization in the noninvasive ventilation arm, n (%) | 72 (48%) | 38 (53%) | 35 (46%) | 0.661 |
| Respiratory rate, breaths/min | 29 ± 7.7 | 28 ±7.4 | 28 ±7.2 | 0.810 |
| pH, units | 7.44 ± 0.08 | 7.43 ± 0.08 | 7.44 ± 0.06 | 0.633 |
| PaO_2_/FiO_2_, mm Hg | 179 ± 93 | 156 ± 82 | 170 ± 84 | 0.242 |
| PaCO_2_, mm Hg | 34 ± 6 | 34 ± 7 | 33 ± 6 | **0.002** |
| Discomfort score, mm | 43 ± 28 | 43 ± 27 | 36 ± 28 | 0.236 |
| **Change between inclusion and H1, n (%)** |  |  |  |  |
| Increased respiratory rate | 50 (41%) | 23 (32%) | 16 (22%) | 0.173 |
| Increased PaO_2_/FiO_2_ | 75 (60%) | 38 (66%) | 34 (59%) | 0.690 |
| Increased PaCO_2_ | 53 (42%) | 26 (45%) | 25 ((42%) | 0.924 |
| Increased discomfort score | 46 (31%) | 30 (42%) | 18 (23%) | 0.300 |

ECOG: Eastern Cooperative Oncology Group; SOFA: Sequential organ failure assessment; PaO_2_: partial pressure of arterial oxygen; FiO_2_: fraction of inspired oxygen, PaCO_2_: partial pressure of arterial carbon dioxide; H1: 1 hour after randomization; ICU: intensive care unit

**Additional Table 2. Baseline characteristics of patients in whom ECOG data at 6 months was missing.**

| **Baseline patient characteristics** | **N=3** |
| --- | --- |
| Age, y | 66 [51-67] |
| Sex, male, n (%) | 3 (100%) |
| Body mass index, kg/m^2^ | 24 [22-32] |
| Simplified Acute Physiology score II | 49 [42-65] |
| SOFA score | 5 [4-7] |
| ECOG performance status | 0 [0-1] |
| Charlson comorbidity score | 3 [2-6] |
| **Underlying condition**, n (%) |  |
| Type of immunosuppression |  |
| Hematological malignancy | 2 (67%) |
| Solid cancer | 0 (0%) |
| Acquired Immunodeficiency Syndrome | 1 (33%) |

SOFA: Sequential organ failure assessment; ECOG: Eastern Cooperative Oncology Group
